# Inhibitors of the Hydrolytic Enzyme Dimethylarginine Dimethylaminohydrolase (DDAH): Discovery, Synthesis and Development

**DOI:** 10.3390/molecules21050615

**Published:** 2016-05-11

**Authors:** Rhys B. Murphy, Sara Tommasi, Benjamin C. Lewis, Arduino A. Mangoni

**Affiliations:** Department of Clinical Pharmacology, School of Medicine, Flinders University, Bedford Park, Adelaide 5042, Australia; rhys.murphy@flinders.edu.au (R.B.M.); sara.tommasi@flinders.edu.au (S.T.); ben.lewis@flinders.edu.au (B.C.L.)

**Keywords:** dimethylarginine dimethylaminohydrolase, arginine, nitric oxide, asymmetric dimethylarginine, monomethyl arginine, enzyme inhibitors, organic synthesis

## Abstract

Dimethylarginine dimethylaminohydrolase (DDAH) is a highly conserved hydrolytic enzyme found in numerous species, including bacteria, rodents, and humans. In humans, the DDAH-1 isoform is known to metabolize endogenous asymmetric dimethylarginine (ADMA) and monomethyl arginine (l-NMMA), with ADMA proposed to be a putative marker of cardiovascular disease. Current literature reports identify the DDAH family of enzymes as a potential therapeutic target in the regulation of nitric oxide (NO) production, mediated via its biochemical interaction with the nitric oxide synthase (NOS) family of enzymes. Increased DDAH expression and NO production have been linked to multiple pathological conditions, specifically, cancer, neurodegenerative disorders, and septic shock. As such, the discovery, chemical synthesis, and development of DDAH inhibitors as potential drug candidates represent a growing field of interest. This review article summarizes the current knowledge on DDAH inhibition and the derived pharmacokinetic parameters of the main DDAH inhibitors reported in the literature. Furthermore, current methods of development and chemical synthetic pathways are discussed.

## 1. Introduction

Dimethylarginine dimethylaminohydrolase (DDAH) is a modulator of the nitric oxide (NO) pathway and is responsible for the metabolism of methylated arginines [1]. DDAH is conserved throughout many species and has been demonstrated to pre-date evolution of the Nitric Oxide Synthase (*NOS*) family [2], suggesting an alternative function for methylated arginines independent to their role as NOS regulatory molecules. Two different isoforms of human DDAH have been identified, with DDAH-1 and DDAH-2 sharing 62% homology. DDAH-1 is primarily expressed in different areas of the brain [3], the dorsal root ganglia and the spinal dorsal horn [4]. DDAH-2 appears prevalent in pancreatic islet cells [5] and infected tissues [6]. In addition, Altman *et al.* reported a study showing high expression of DDAH-2 in porcine immune tissues [7]. The two isoforms are both expressed in kidney [8,9,10], heart [11,12], adipose tissue [13], placenta and trophoblasts [14], and the liver [15,16,17,18]. Phylogenetic analyses indicate DDAH-1 was generated by duplication of the genomic segment located on chromosome 6 comprising DDAH-2 [2,19]. Although hydrolysis of asymmetrically methylated arginines appears to be the primary function of DDAH-1, alternate roles for both DDAH isoforms are evident when they independently interact with other enzymes [20,21]. For example, DDAH-1 is reported to bind the tumour suppressor protein neurofibromin, resulting in increased phosphorylation of neurofibromin by protein kinase A (PKA) [20] and is additionally reported to regulate the levels of phosphorylated protein kinase B (p-Akt) by increasing Ras activity via a mechanism independent to the NOS pathway [22]. Similarly, DDAH-2 is known to interact with PKA causing the induction of vascular endothelial growth factor (VEGF) via phosphorylation of the Sp1 transcription factor [21].

**Figure 1:**
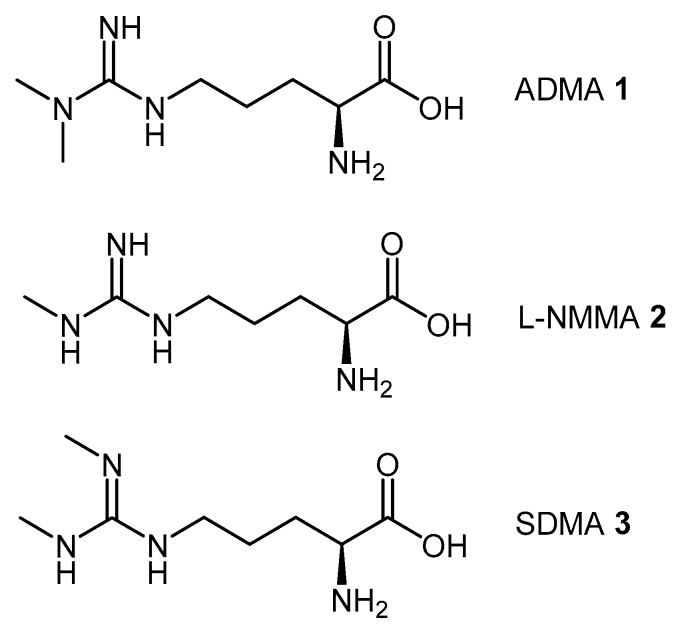
The endogenous methylated arginines, asymmetric dimethylarginine (ADMA **1**), monomethylarginine (l-NMMA **2**), and symmetric dimethylarginine (SDMA **3**) act as direct or indirect inhibitors of the NOS family of enzymes. ADMA (**1**) and l-NMMA (**2**) are substrates for human DDAH-1.

**Scheme 1:**
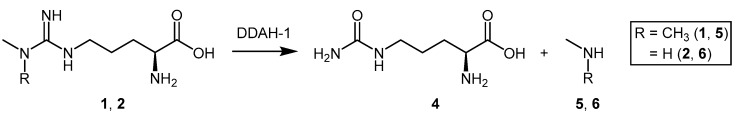
ADMA and l-NMMA are substrates for DDAH-1 and are converted to l-citrulline (**4**) and an amine species **5** or **6**.

Asymmetric dimethylarginine (ADMA, **1**), monomethylarginine (l-NMMA, **2**), and symmetric dimethylarginine (SDMA, **3**) are endogenous methylated arginines (Figure 1). They are formed by two biochemical processes: (1) the initial post-translational methylation of protein arginine residues which is catalyzed by the protein arginine methyltransferases (PRMT’s); and (2) the subsequent release from their respective methylated protein(s) by proteolysis [23]. Once released into the cytosol, transport and efflux of the methylated arginines is mediated by the y^+^ cationic amino acid (CAT-1) transporter [24]. ADMA and l-NMMA inhibit all members of the NOS family of enzymes [25,26,27,28,29,30], while SDMA competes with l-arginine, a NOS substrate, for transport by the y^+^ cation transporter [31]. Different enzymes play a role in the metabolism of methylated arginines, particularly alanine-glyoxylate aminotransferase 2 (AGXT2) [32,33,34]. However, ADMA and l-NMMA, but not SDMA, are primarily converted by DDAH-1 to l-citrulline (**4**) and dimethylamine (**5**) or monomethylamine (**6**), respectively [35] (Scheme 1).

The role of ADMA accumulation and impaired DDAH expression and/or activity in the pathophysiology of several diseases is well documented [36,37,38,39,40,41,42,43,44]. Increased plasma or serum ADMA concentrations have been associated with coronary events [45,46,47], renal disease [29,37], atherosclerosis [48], and cerebrovascular disease [49,50]. Up-regulation of DDAH expression and activity might, therefore, represent a good strategy in the treatment of the aforementioned pathologies to increase ADMA metabolism and lower its plasma concentrations.

In contrast, reduced ADMA concentrations have been demonstrated in other disease states, such as amyotrophic lateral sclerosis [51], multiple sclerosis [43], Alzheimer’s disease and dementia [52,53]. ADMA has also been shown to have neuroprotective effects in a model of Parkinson’s disease [54]. Furthermore, DDAH overexpression in tumours enhances their metastatic potential [42,55,56]. Several reports also show that inhibition of NO synthesis results in significant anti-angiogenic effects both *in vitro* and *in vivo*, with potential beneficial effects in cancer [57,58,59,60,61]. Moreover, excessive NO concentrations play a key role in the pathophysiology of septic shock [62,63,64,65] (an early therapeutic approach for the treatment of septic shock involved the administration of 546C88 (l-NMMA) to inhibit NOS and reduce NO synthesis, however the increase in mortality in patients receiving l-NMMA caused the termination of the clinical trial in phase III [66]. Further analysis of the data revealed that mortality was associated with elevated l-NMMA concentrations; conversely, lower l-NMMA concentrations were associated with improved survival [36]. In this context a more controlled, indirect modulation of methylated arginine concentrations through pharmacological DDAH-1 inhibition (DDAH-2 knockout affects outcome in models of polymicrobial sepsis [44]) might represent an alternative strategy, and appears particularly promising in a rat model of septic shock [67]). In these circumstances, DDAH inhibition could exert a controlled inhibition of the NO synthesis by increasing ADMA concentrations and offering a new approach in the treatment of these diseases.

Therefore, alterations in DDAH and NOS activity might exert either beneficial or toxic effects, according to specific experimental models and disease conditions. The potential of DDAH as a molecular target in the treatment of these pathological conditions has led several groups to study the endogenous mechanisms involved in the modulation of the expression and activity of human DDAH-1 and DDAH-2 [68]. The elucidation of these mechanisms may identify new strategies for the pharmacological activation or inhibition of these enzymes to restore physiological homeostasis after pathological imbalance. Since the discovery of the DDAH family of enzymes in 1987 [35], a number of research groups have identified inhibitors with the aim of developing such therapeutics. While no ‘selective’ DDAH inhibitor has entered clinical trials, numerous compounds have been synthesized that act as prototypic *in vitro* inhibitors. Herein, we discuss all published DDAH inhibitors according to their chemical structure (‘substrate-like’ and ‘non-substrate-like’), and summarize the details of their synthetic pathways as well as how they alter the pharmacokinetic parameters for l-citrulline formation by DDAH.

## 2. Endogenous Modulation of DDAH Activity

The production of specific gene products, both in terms of RNA and proteins, is physiologically regulated by a plethora of mechanisms and can be triggered or silenced by various stimuli and factors. Of particular note, DDAH activity is reported to be inhibited by divalent transition metals [69,70] and it is known that elevated zinc(II) concentrations are associated with oxidative stress in numerous pathological conditions [71,72,73].

*S*-Nitrosylation of the enzyme’s cysteine residues is a further important post-translational regulatory mechanism [74] and is generally associated with increased expression of inducible NOS (iNOS). The induction of iNOS generates excessive NO that can bind covalently (but reversibly) to the thiol (SH) moiety of each DDAH cysteine [74]. The apparent *S*-nitrosylation of the catalytic DDAH cysteine residue results in DDAH inhibition. This in turn causes the subsequent accumulation of DDAH substrates, ADMA and l-NMMA, which eventually reach concentrations that reversibly inhibit the NOS enzymes [75].

Oxidative stress additionally modulates DDAH activity. Factors associated with oxidative stress, including shear stress [76], tumour necrosis factor α (TNF-α), oxidized low density lipoprotein (LDL) [76], and homocysteine [77] each suppresses DDAH expression and activity. In contrast, antioxidant mediators such as probucol [78], estradiol [79], and interleukin-1β (IL-1β) [80] up-regulate DDAH expression. Moreover, nicotine and cigarette smoking have been shown to decrease DDAH expression and activity in endothelial cells, with consequential accumulation of ADMA, NOS inhibition, and endothelial damage [81,82]. Furthermore, it is known that selective down-regulation of DDAH-1 and up-regulation of DDAH-2 occurs when angiotensin II binds to the type 1 angiotensin receptor (AT_1_-R), linking DDAH transcription to angiotensin-induced inflammation [8]. In addition, epigenetic modifications are known to alter DDAH-2 regulation at the promoter. For example, DNA hypermethylation has the ability to suppress DDAH-2 transcription, while histone acetylation has the ability to up-regulate DDAH-2 transcription [83].

## 3. Pharmacological Induction of DDAH

As a result of diminished DDAH activity and/or accumulation of ADMA and l-NMMA in various disease states, several studies have investigated the effects of restoring or increasing *DDAH* expression. Statin treatment is associated with a significant reduction in ADMA concentrations [84,85,86], and an increase in both DDAH-1 and DDAH-2 expression and activity [87,88,89]. Another class of compounds enhancing ADMA metabolism via DDAH up-regulation is represented by the farnesoid X receptor (FXR) agonists. GW4064 upregulates both DDAH-1 and the cationic transporter *CAT-1*, leading to increased ADMA cellular uptake and metabolism, and decreased serum ADMA concentrations [15,90]. Another FXR agonist, INT-747, can increase DDAH-1 expression [91,92], however this is not associated with significant changes in ADMA and NO concentrations [91]. The lipid lowering drug probucol also lowers ADMA concentrations [93] via up-regulation of both DDAH expression and activity, and down-regulation of PRMT-1 expression [78,94]. Another lipid lowering drug, fenofibrate, decreases ADMA concentrations by restoring DDAH activity in conditions of oxidative stress [95]. Additionally, the oral antidiabetic drug pioglitazone and the β_1_ receptor blocker nebivolol both decrease serum ADMA concentrations by increasing DDAH-2 expression [96,97,98,99].

## 4. Pharmacological Inhibition of DDAH

Contrary to the comments in Section 3 above, there is a growing body of evidence to suggest that the modulation of DDAH enzyme activity may be utilized in conditions characterized by excessive NO production [36,67]. The remainder of this review will focus on highlighting the current knowledge associated with the main DDAH inhibitors reported in the literature to date.

### 4.1. Substrate-Like Inhibitors

#### 4.1.1. *S*-Nitroso-l-Homocysteine (HcyNO)

**Scheme 2:**
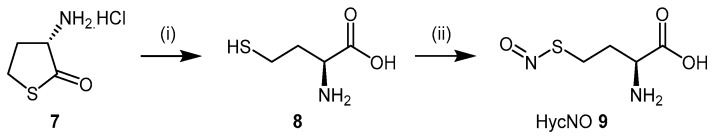
Synthetic scheme for generating HcyNO (**9**). *Reagents and Conditions*: (**i**) 5 M NaOH, 37 °C, 5 min, 96%; (**ii**) NaNO_2_, 2 M HCl, 37 °C, 15 min, >99%.

Since the identification of *S*-nitrosylation as an important mechanism of DDAH regulation, the discovery of l-homocysteine’s ability to inhibit DDAH-1 led to the development of *S*-nitroso-l-homocysteine (HcyNO, **9**), an endogenous *S*-nitrosothiol and a source of endogenous NO [100,101,102]. Synthesis and characterisation of HcyNO as a bovine DDAH inhibitor was reported by Knipp *et al.* (2005) [102]. HcyNO was synthesized in near quantitative yield in a two-step reaction (Scheme 2) via alkaline hydrolysis of commercially available l-homocysteine thiolactone hydrochloride (**7**) to l-homocysteine (**8**) [103], followed by *S*-nitrosylation of l-homocysteine with sodium nitrite (NaNO_2_) and acidification to afford HcyNO (**9**) [104]. Alternatively, *S*-nitrosylation can be undertaken directly on commercially available l-homocysteine (**8**). Mild reaction conditions are required, and excellent yields make HcyNO simple to prepare on a large scale.

**Scheme 3:**
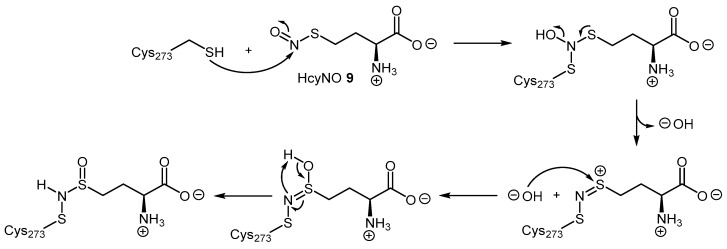
Schematic of the reaction mechanism proposed by Knipp *et al.* [102] for the covalent inhibition of DDAH-1 by HcyNO (**9**) at Cys273. Adapted with permission from [102]. Copyright 2005 American Chemical Society.

HcyNO irreversibly inhibits DDAH by covalently binding to the putative catalytic cysteine with an inhibitory constant (*K*_i_) of 690 μM [105]. Mass spectrometry analyses using different isotopic forms of *S*-nitroso-l-homocysteine (Hcy^15^NO and HcyN^18^O) and oxygen-18 labelled water (H_2_^18^O) demonstrated that HcyNO forms a *N*-thiosulfoximide adduct with cysteine 273 (Cys273) of bovine DDAH-1. The authors’ proposed mechanism of inhibition requires the lone pair of electrons of the Cys273 SH to attack the *S*-nitroso group in HcyNO to eliminate a hydroxyl anion (Scheme 3). This mechanism is likely stabilized by neighbouring amino acids, namely His172. A further hydroxyl anion originating from water attacks the cationic sulfur of the conjugated intermediate, and subsequently rearranges to give the covalent thiosulfoximide adduct [102].

#### 4.1.2. 2-Chloroacetamidine and *N*-But-3-ynyl-2-chloroacetamidine

**Scheme 4:**
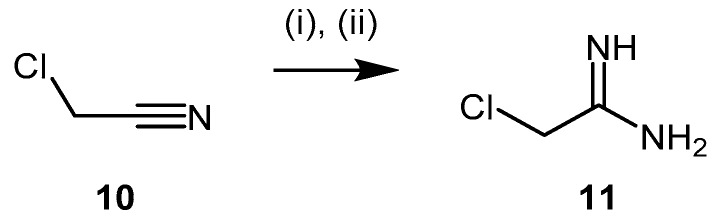
Synthetic scheme for generating 2-chloroacetamidine (**11**) (the conditions described afford the hydrochloride salt). *Reagents and Conditions*: (**i**) NaOMe, dry MeOH, overnight; (**ii**) AcOH, NH_4_Cl, reflux, 6 h, 89%.

Stone *et al.* [106] identified 2-chloroacetamidine (**11**) as a nonspecific inhibitor of two enzymes of the amidinotransferases superfamily, *Pseudomonas aeruginosa* DDAH (*Pa*DDAH) and human peptydilarginine deaminase (PAD). 2-Chloroacetamidine (**11**, Scheme 4) is a commercially available compound bearing a 2-chloromethylene chain and an amidinium group, which partially resembles arginine. It can be synthesized via reaction of chloroacetonitrile (**10**) with sodium methoxide followed by treatment with ammonium chloride [107,108]. 2-Chloroacetamidine acts as a weak irreversible amidinotransferase inhibitor with greater affinity for *Pa*DDAH, relative to PAD4 (Table 1). Data acquired by electrospray ionization mass spectrometry revealed the formation of an irreversible thiouronium-enzyme adduct whereby the loss of chlorine from the inhibitor is observed [106].

**Scheme 5:**
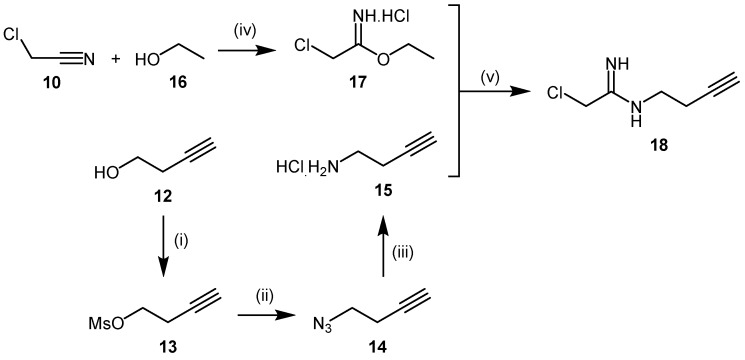
Synthetic scheme for generating the DDAH-1 biochemical probe *N*-but-3-ynyl-2-chloro-acetamidine (**18**). *Reagents and Conditions*: (**i**) DCM, TEA, DMAP, MsCl, 0 °C for 2 h then 25 °C for 3 h, 62%; (**ii**) anhydrous DMF, NaN_3_, 70 °C, 3.5 h; (**iii**) Et_2_O, Ph_3_P, 0 °C for 2 h, addition of H_2_O, then 25 °C for 20 h, 49% (two steps); (**iv**) dry HCl_(g)_ at 0 °C for 1 h, stop HCl_(g)_, then 0 °C for 4 h and RT overnight 48%–76%; (**v**) H_2_O, pH = 10, 0 °C for 3 h, 25 °C for 2 h, neutralized, RT for 1.5 days, 62%. Adapted with permission from [109]. Copyright 2009 American Chemical Society.

The addition of an *N*-but-3-ynyl substituent to the acetamidine group of 2-chloroacetamidine led to formation of the human DDAH-1 probe, *N*-but-3-ynyl-2-chloro-acetamidine (**18**, Scheme 5) [109]. *N*-But-3-ynyl-2-chloro-acetamidine binds within the putative active-site, while the *N*-alkynyl substituent is reacted with biotin-PEO_3_-azide under copper catalysis via click chemistry. Successful conjugation can be easily detected in immunoblots using an anti-biotin antibody [109]. Synthesis of the *N*-but-3-ynyl-2-chloro-acetamidine probe was achieved in five steps using two modules (Scheme 5). Amine module **15** was prepared in three steps: activation of the terminal hydroxyl group of 3-butyn-1-ol (**12**) through mesylation to afford **13** in 62% yield, followed by conversion to amine **15** via azide intermediate **14** in 49% yield across two steps. The imidic ester module **17** was obtained in moderate yields (approximately 48%) by reacting chloroacetonitrile (**10**) with anhydrous ethanol (**16**) in the presence of dry hydrochloric acid gas. Final coupling between amine **15** and the imidic ester **17** proceeds under basic aqueous conditions to afford probe *N-*but-3-ynyl-2-chloro-acetamidine (**18**) in reasonable yields (approximately 63%). To date, no data are available regarding the selectivity of *N*-but-3-ynyl-2-chloro-acetamidine for human DDAH-1 and the potential cross reactivity of this probe with *Pa*DDAH.

#### 4.1.3. (*S*)-2-Amino-4-(3-Methylguanidino)butanoic Acid Analogues

**Scheme 6:**
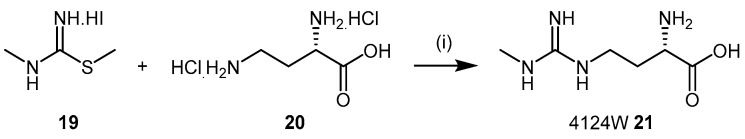
Synthetic scheme for generating (*S*)-2-amino-4-(3-methylguanidino)butanoic acid (4124W), as reported by Ueda *et al.* [80] using the methods of Ogawa *et al.* [32] and Corbin *et al.* [111]. *Reagents and Conditions*: (**i**) (1) 2,4-diamino-*n*-butyric acid dihydrochloride (**20**), copper acetate, 1:1 NH_4_OH/H_2_O; (2) *N*,*S*-dimethylthiopseudouronium iodide (**19**) RT, 24 h; (3) Dowex 50WX4 (NH_4_^+^ form), 4124W (**21**) was crystallized as the hydrochloride salt.

Screening experiments with a library comprising 26 analogues of ADMA (**1**) and l-NMMA (**2**) identified (*S*)-2-amino-4-(3-methylguanidino)butanoic acid (4124W, **21**), as a mammalian DDAH inhibitor [110]. The original synthesis of 4124W (**21**), first described by Ueda *et al.* in 2003 [80] is shown in Scheme 6, and was achieved by coupling *N*,*S*-dimethylthiopseudouronium iodide (**19**) with the copper complex of 2,4-diamino-*n*-butyric acid dihydrochloride (**20**) [32,111]. 4124W (**21**) was finally crystallized as the hydrochloride salt.

**Scheme 7:**
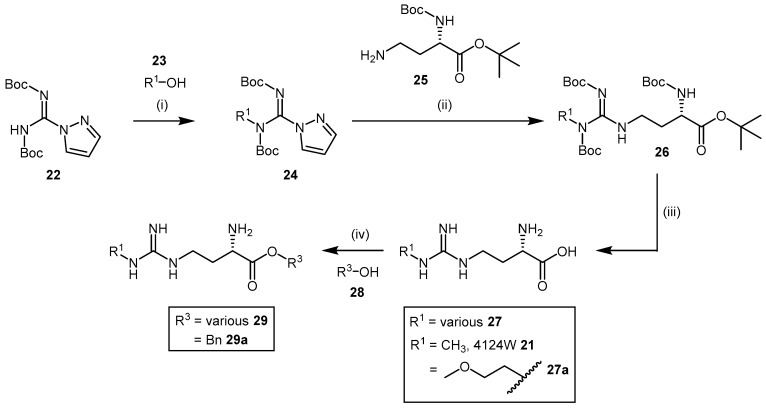
Synthetic scheme for generating (*S*)-2-amino-4-(3-methylguanidino)butanoic acid analogues. *Reagents and Conditions*: (**i**) DEAD, PPh_3_, THF, 0 °C-RT, 3–16 h; (**ii**) DIPEA, CH_3_CN, RT, 24 h; (**iii**) 4M HCl/1,4-dioxane, RT, 24–72 h, 12%–67% overall; (**iv**) SOCl_2_, 0 °C for 30 min, reflux for 1 h, RT overnight, 44%–84%. Adapted with permission from [112]. Copyright 2005 American Chemical Society.

High chemical and structural similarity between 4124W and the DDAH substrate l-NMMA exists (compound **21**, Scheme 6, and compound **2**, Figure 1), and although exhibiting weak DDAH inhibition (IC_50_ = 416 μM) [110], this prompted Rossiter *et al.* [112] to synthesize a further 34 compounds based on the 4124W structure. Furthermore, Rossiter *et al.* [112] reported an alternative synthetic approach, which although requiring three steps, is synthetically versatile and was applied to develop their library of 4124W analogues. Rossiter’s strategy utilized protected intermediates to avoid complications associated with the purification of polar amino acids. Intermediate **24** (Scheme 7) is generated from commercially available *N*,*N′*-bis-*tert*-butoxycarbonylpyrazole-1*H*-carbox-amidine (**22**) by Mitsunobu reaction with alcohol **23** containing the desired R^1^ group, followed by reaction with (*S*)-*tert*-butyl 4-amino-2-((*tert*-butoxycarbonyl)amino)butanoate (**25**) to afford the protected amino acid analogue **26** (Scheme 7). Cleavage of the Boc protecting groups is achieved with a 4 M solution of HCl in 1,4-dioxane and produced 4124W (**21**) in moderate yields (approximately 44%) or analogues **27** in moderate to good yields (12%–67%).

**Scheme 8:**
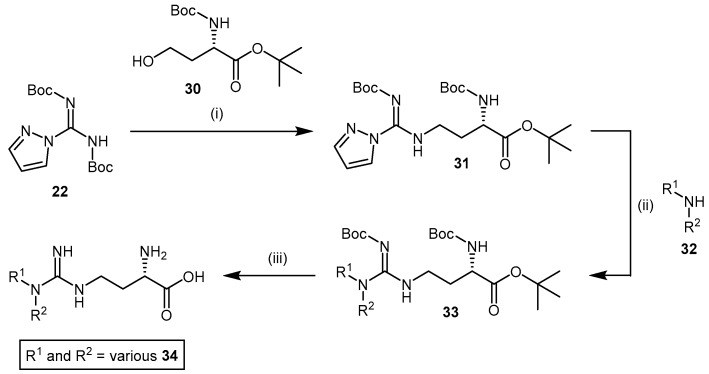
Synthetic scheme for generating *N*,*N*-disubstituted alkylguanidinobutanoic acid and *N*-arylguanidine analogues. *Reagents and Conditions*: (**i**) DEAD, Ph_3_P, THF, 0 °C-RT, 3–16 h; (**ii**) DIPEA, CH_3_CN, RT, 24 h; (**iii**) 4 M HCl/1,4-dioxane, 24–72 h, 0.29%–41% overall. Adapted with permission from [112]. Copyright 2005 American Chemical Society.

Additionally, Rossiter *et al.* developed a synthetic method for *N,N*-disubstituted alkylguanidinobutanoic acids and *N*-arylguanidines (Scheme 8). This involved reacting *N,N’*-bis-*tert*-butoxycarbonylpyrazole-1*H*-carboxamidine (**22**) with Boc-homoserine-*tert*-butyl ester (**30**), whereby the pyrazole group in the intermediate **31** is displaced by the reaction with amine **32** containing the desired R^1^ and R^2^ groups. 

Deprotection of the amino acid analogues **33** is carried out using the same acidic conditions described in the synthesis of Rossiter’s 4124W analogues in Scheme 7 and provides analogues **34** in low to moderate yields (0.29%–41%). Each analogue’s inhibitory potential was assessed based on the reduced conversion of [^14^C]-NMMA to [^14^C]-citrulline by rat DDAH. Screening experiments revealed *N*-monosubstitution to be more effective than disubstitution, and that a R^1^ substituent comprising a *N*-2-methoxyethyl group increased inhibitor binding affinity (viz. compound **27a**; IC_50_ = 189 μM; Scheme 7). Furthermore, a series of *N*-2-methoxyethyl guanidinobutanoate esters [112] was synthesized via thionyl chloride promoted esterification with alcohol containing the desired R^3^ group (Scheme 7). The generated benzyl ester **29a** resulted in substantial improvement in DDAH-1 inhibition (IC_50_ = 27 μM). A series of amide analogues based on structures **29** (structures omitted) exhibited poor DDAH-1 inhibitory potentials.

#### 4.1.4. *N*^G^-Substituted-l-Arginine Analogues

**Scheme 9:**
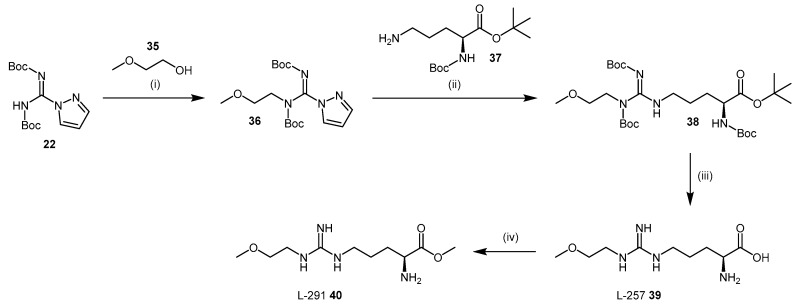
Synthetic scheme for generating L-257 (**39**) and L-291 (**40**). *Reagents and Conditions*: (**i**) DEAD, Ph_3_P, THF, 0 °C-RT, 3–16 h; (**ii**) DIPEA, CH_3_CN, RT, 24 h; (**iii**) 4 M HCl/1,4-dioxane, 24–72 h, 44% overall; (**iv**) SOCl_2_, 0 °C for 30 min, reflux for 1 h, RT overnight, 80%. Adapted with permission from [112]. Copyright 2005 American Chemical Society.

Rossiter *et al.* [112] also synthesized a library of *N*-substituted arginine analogues. A significant increase in inhibitor binding affinity was obtained for *N^G^*-(2-methoxyethyl)-l-arginine (commonly known as L-257 (compound **39**; IC_50_ = 22 μM; Scheme 9) and its corresponding methyl ester (also known as L-291 (compound **40**; IC_50_ = 20 μM; Scheme 9), relative to compound **27a** (Scheme 7) that contains one less carbon within the main-chain. Interestingly, the benzyl ester of L-257 did not increase DDAH-1 inhibition as was observed with compound **29a**. These data suggest that arginine analogues may bind differently within the putative DDAH-1 active-site. Moreover, neither the corresponding methyl amide of L-257, nor the (*R*)-isomer of L-257 (structures omitted) resulted in DDAH-1 inhibition.

The synthesis of L-257 (**39**) is represented in Scheme 9. The reaction sequence follows a similar approach to that described in the synthesis of the (*S*)-2-amino-4-(3-methylguanidino)butanoic acid analogues, using Boc protected amino acid analogues. Firstly, 2-methoxyethyl pyrazole carboxamidine (**36**) is prepared under Mitsunobu conditions [113] from 2-methoxyethanol (**35**) and *N,N′*-bis-*tert*-butoxycarbonylpyrazole-1*H*-carboxamidine (**22**). This intermediate **36** is reacted with Boc-Orn-OBu*^t^* (**37**) to introduce the *N*-alkyl-guanidino group to compound **38**. Final deprotection of **38** with 4 M HCl in 1,4-dioxane afforded L-257 (**39**) in 44% overall yield. L-257 can be subsequently esterified to afford L-291 (**40**) in yields of approximately 80%.

Both L-257 (**39**) and L-291 (**40**) inhibit DDAH-1 *in vivo* [112]. They are non-cytotoxic and selective for DDAH-1 against the three main isoforms of NOS [112,114] and arginase [115], an enzyme responsible for the metabolism of l-arginine into urea and ornithine [112,114,115]. L-257 (**39**) was utilized by Kotthaus *et al.* [114] as a validated active ‘parent’ compound for determining viable lead compounds, and to obtain initial structure-activity relationships (SARs) for those leads. In particular, the role of the *N*-substituent in DDAH inhibition was investigated as the guanidino moiety is considered to be important in the proposed catalytic mechanism and binding to the enzyme [116]. In the work undertaken by Kotthaus *et al*., the *N^G^*-(2-methoxyethyl) side-chain was varied along with the degree of guanidine substitution. Similarly, Tommasi *et al.* synthesized further L-257 derivatives with differing side-chains [117]. In both studies, the *N^G^*-(2-methoxyethyl) side-chain conferred inhibitors with activity toward DDAH-1. *N*-monosubstituted examples are represented in Table 2.

#### 4.1.5. *N*^G^-(2-Methoxyethyl)-l-Arginine Carboxylate Bioisosteres

**Scheme 10:**
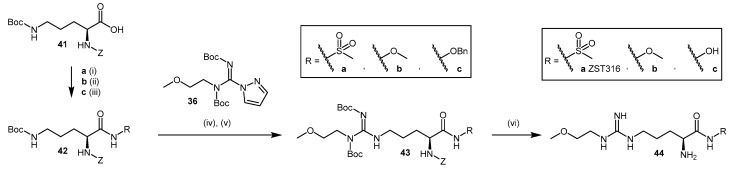
Synthetic scheme for generating *N*^G^-(2-methoxyethyl)-l-arginine carboxylate bioisosteres. *Reagents and Conditions*: (**i**) CDI, DBU, methanesulfonamide, THF, RT, 7 h, 51%; (**ii**) HATU, DIPEA, *O*-methylhydroxylamine hydrochloride, THF, 0 °C-RT, 16 h, 86%; (**iii**) HATU, DIPEA, *O*-benzylhydroxylamine, THF, 0 °C-RT, 16 h, 57%; (**iv**) 4 M HCl/1,4-dioxane, DCM, RT, 30 min, 99%; (**v**) DIPEA, DCM, RT, 24 h, **43a** 42%, **43b** 22%, **43c** 56%; (**vi**) TFMSA/TFA, RT, 1 h, 99%. Reproduced in part from [117] with permission of the Royal Society of Chemistry.

**Scheme 11:**
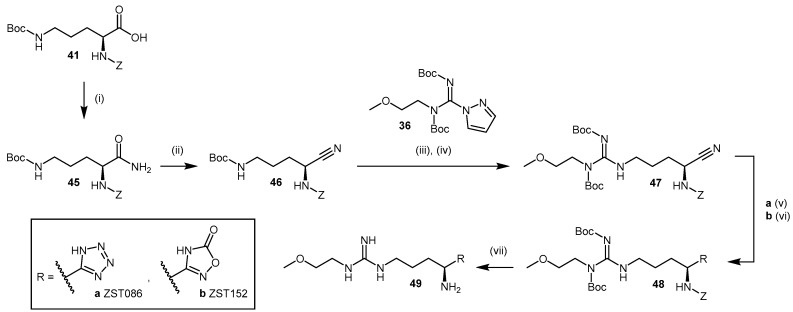
Synthetic scheme for generating *N*^G^-(2-methoxyethyl)-l-arginine carboxylate bioisosteres. *Reagents and Conditions*: (**i**) *i*-BuCOCl, *N*-methylmorpholine, NH_4_OH (30%), THF, −10 °C-RT, 3 h, 99%; (**ii**) TFAA, TEA, THF, 0 °C-RT, 16 h, 47%; (**iii**) 4 M HCl/1,4-dioxane, DCM, RT, 30 min, 99%; (**iv**) DIPEA, DCM, RT, 24 h, 40%; (**v**) NaN_3_, ZnBr_2_, H_2_O/2-propanol, reflux, 16 h, 30%; (**vi**) (1) NH_2_OH·HCl, NaHCO_3_, DMSO, reflux, 16 h; (2) CDI, DBU, THF, reflux, 30 min, 73%; (**vii**) TFMSA/TFA, DCM, RT, 30 min, 99%. Reproduced in part from [117] with permission of the Royal Society of Chemistry.

The use of bioisosteric replacements is a well-known strategy adopted by medicinal chemists to improve the pharmacokinetic and pharmacodynamic properties of certain compounds, including but not limited to inhibition potency, lipophilicity, pK_a_, and metabolic stability [118]. Tommasi *et al.* [117] reported the synthesis and pharmacological evaluation of five carboxylate bioisosteres of L-257 (compounds **44a**–**c**; Scheme 10, **49a**–**b**; Scheme 11). In compounds **44a**–**c** (Scheme 10), the carboxylic acid moiety is modified by introducing an acyl methyl sulfonamide **44a** (2-amino-5-(3-(2-methoxyethyl)guanidino)-*N*-(methylsulfonyl)-pentanamide; ZST316), an *O*-methyl-hydroxamate **44b** and a hydroxamate **44c**. The replacement is introduced on Z-ornithine(Boc)-OH (**41**) in moderate to good yields (51%–86%) to afford **42**, followed by the quantitative cleavage of Boc protected **42** with 4 M HCl in 1,4-dioxane. Guanidination of **42** with 2-methoxyethylpyrazole carboxamidine (**36**) is undertaken as described by Rossiter *et al.* [112] (see Section 4.1.3 and Section 4.1.4, Scheme 7 and Scheme 9). Deprotection of the Boc- and Z-appended arginine derivatives **43a**–**c** is achieved in a single step using TFMSA and TFA [117,119] and produces compounds **44a**–**c**.

Bioisosteric replacement of the carboxylic acid moiety in L-257 with *O*-methyl hydroxamate **44b** and hydroxamate **44c** resulted in a decrease in DDAH-1 inhibition (IC_50_ = 230 and 131 μM, respectively). By contrast, introduction of an acyl methyl sulfonamidic moiety in ZST316 (**44a**) led to significant improvements in pharmacokinetic parameters (98% inhibition at 1 mM, IC_50_ = 3 μM and *K*_i_ = 1 μM). Compound ZST316 (**44a**) is the most potent human DDAH-1 inhibitor with ‘substrate-like’ structure reported in the literature to date.

Other noteworthy inhibitors in this series include the tetrazole 1-[4-amino-4-(1*H*-tetrazol-5-yl)butyl]-3-(2-methoxyethyl)guanidine (ZST086, **49a**, Scheme 11) and the 5-oxo-1,2,4-oxadiazole 1-[4-amino-4-(5-oxo-4,5-dihydro-1,2,4-oxadiazol-3-yl)butyl]-3-(2-methoxyethyl)guanidine (ZST152, **49b**, Scheme 11) [117]. The synthesis of these inhibitors with azole bioisosteric groups is achieved via conversion of the terminal carboxylic acid in **41** to primary amide **45** in quantitative yields using isobutylchloroformate, *N*-methylmorpholine, and aqueous ammonia. Primary amide **45** is then converted to nitrile **46** using trifluoroacetic anhydride and triethylamine in 47% yield. Compound **46** is guanylated [112] (see Section 4.1.3 and Section 4.1.4, Scheme 7 and Scheme 9) to afford compound **47**, from which the heterocycle is constructed from the nitrile. The tetrazole is introduced by treating nitrile **47** with sodium azide in the presence of zinc(II) bromide to afford compound **48a**, using the click chemistry procedure reported by Demko and Sharpless [120]. Although the use of zinc(II) bromide in the synthesis of Boc-amino tetrazoles derived from α-amino acids has been reported [121], the use of a Lewis acid may result in partial deprotection of compound **48a** during the reaction. Ammonium chloride represents an alternative reagent [122]. The 5-oxo-1,2,4-oxadiazole **48b** is synthesized from nitrile **47** using hydroxylamine hydrochloride and sodium bicarbonate in DMSO, followed by treatment with CDI and DBU [123]. Acidic deprotection of **48a**–**b** is achieved as described previously [117,119]. Despite obtaining lower yields for heterocyclic adducts **49a**–**b** (7%–13%) relative to bioisosteres **44a**–**c** (19%–32%), the inhibitory potential of 5-oxo-1,2,4-oxadiazole ZST152 (**49b**) was significant (95% at 1 mM, IC_50_ = 18 μM, *K*_i_ = 7 μM), while tetrazole ZST086 (**49a**) displayed 91% inhibition at 1 mM, with IC_50_ = 34 μM and *K*_i_ = 14 μM [117].

#### 4.1.6. *N^5^*-(1-Iminoalk(en)yl)-l-Ornithine Derivatives

**Scheme 12:**
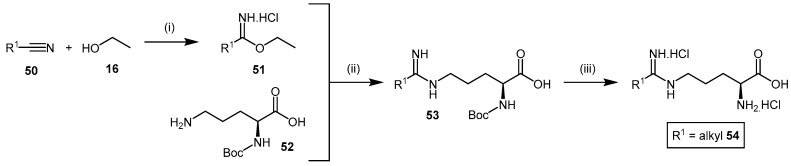
Synthetic scheme for generating *N*^5^-(1-iminoalk(en)yl)-l-ornithine derivatives. *Reagents and Conditions*: (**i**) dry HCl_(g)_ at 0 °C for 1 h, stop HCl_(g)_, then 0 °C for 4 h and RT overnight; (**ii**) H_2_O, 5 °C, pH = 10, 1.5 h, adjust pH = 7, overnight, Dowex 50WX8-400 (H_2_O then 10% aqueous pyridine); (**iii**) 6 M HCl, EtOAc, 84%–94% from *N*^α^-Boc-l-ornithine (**52**). Adapted with permission from [127]. Copyright 1999 American Chemical Society.

**Figure 2:**
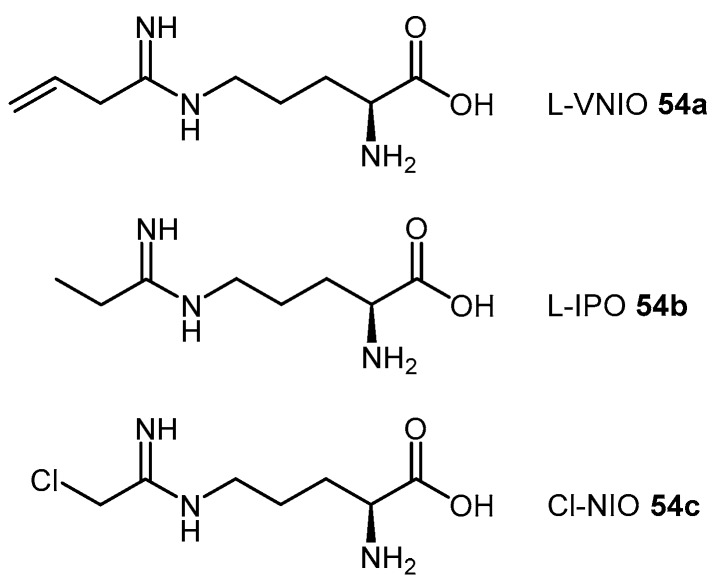
*N*^5^-(1-Iminoalk(en)yl)-l-ornithine derivatives, L-VNIO (**54a**), L-IPO (**54b**), and Cl-NIO (**54c**), as synthesized by Kotthaus *et al.* [114] and Wang *et al.* [128,129].

Amidines are commonly known bioisosteres of the guanidine moiety [124]. *N^5^*-(1-iminoalk(en)yl)-l-ornithines such as *N*^5^-(1-imino-3-butenyl)-l-ornithine (L-VNIO, **54a**, Figure 2), are reported to inhibit NOS by competing with l-arginine for binding at the enzyme catalytic-site [125,126,127]. Therefore, derivatives of this class of compounds have been extensively studied as DDAH inhibitors. The synthetic approach to afford *N^5^*-(1-iminoalk(en)yl)-l-ornithine derivatives **54** (Scheme 12) [127,128], utilized a similar approach to the *N*-but-3-ynyl-2-chloro-acetamidine probe (**18**) (see Section 4.1.2, Scheme 5) [109]. The R^1^ furnished nitrile **50** is converted to its corresponding ethyl imidic ester **51** by bubbling dry hydrochloric acid gas through a solution of anhydrous ethanol (**16**). The ethyl imidic ester **51** is then reacted with *N*^α^-Boc-l-ornithine (**52**) to afford the protected adduct **53**. Deprotection is obtained by treatment with 6 M HCl to achieve the *N*^5^-(1-iminoalk(en)yl)-l-ornithine **54** in excellent overall yields (84%–94%).

The significant structural similarity of the *N*^5^-(1-iminoalk(en)yl)-l-ornithine derivatives with DDAH substrates, ADMA (**1**) and L-NMMA (**2**) led different groups to study this class of compounds as potential DDAH inhibitors [114,128]. Kotthaus *et al.* [114] examined five *N^5^*-(1-iminoalk(en)yl)-l-ornithines with varying side-chain lengths and bond saturation for their ability to inhibit l-citrulline formation by human DDAH-1 using both high performance liquid chromatography and colorimetry. L-VNIO (**54a**, Figure 2) emerged as the best DDAH-1 inhibitor of this series of derivatives (97% inhibition at 1 mM, IC_50_ = 13 μM, *K*_i_ = 2 μM). However, increasing the main-chain length by one carbon atom from ornithine to lysine reduced its DDAH inhibitory potential.

Wang *et al.* [128] investigated this class of compounds as dual DDAH-1 and NOS inhibitors, reporting the synthesis and kinetic parameters for four *N^5^*-(1-iminoalkyl)-l-ornithines. *N*^5^-(1-Iminopropyl)-l-ornithine (L-IPO **54b**, Figure 2) was identified as the best compound exhibiting inhibition of both human DDAH-1 (*K*_i_ = 52 μM) and rat nNOS (*K*_i_ = 3 μM). Wang *et al.* [129] additionally developed an irreversible human DDAH-1 inhibitor, *N^5^*-(1-imino-2-chloroethyl)-l-ornithine (Cl-NIO, **54c**, Figure 2), whose chemical structure reflects a combination of both L-IPO (**54b**) and 2-chloroacetamidine (**11**). The synthesis of Cl-NIO (**54c**) is similar to that described for the *N*^5^-(1-iminoalk(en)yl)-l-ornithine derivatives (Scheme 12). Here, *N*^α^-Boc-l-ornithine is reacted with 2-chloro-1-ethoxyethanimine, and the intermediate obtained is deprotected with 4.6 M HCl in dioxane to generate Cl-NIO (**54c**, Figure 2) in low yields (10%). The inhibition potency of Cl-NIO (**54c**) was assessed via colorimetric assay and exhibited *K*_i_ = 1.3 μM. In summary, several of the *N^5^*-(1-iminoalk(en)yl)-l-ornithine derivatives (**54a**–**c**, Figure 2) are potent human DDAH-1 inhibitors. Off-target effects on the broader NO pathway (NOSs, arginases, AGTX-2) may either represent an advantage (strong NO suppression), or limit their application (loss of NO physiological functions) as potential therapeutics.

### 4.2. Non-Substrate-Like Inhibitors

#### 4.2.1. Pentafluorophenyl (PFP) Sulfonate Esters

**Scheme 13:**
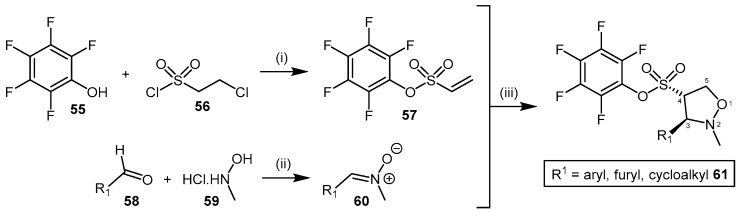
Synthetic scheme for generating PFP sulfonate esters **61**. *Reagents and Conditions*: (**i**) DCM, TEA, 0 °C, 59%; (**ii**) Method A: NaHCO_3_, anhydrous MgSO_4_, DCM, reflux, 24 h, 73%–90% [133]; Method B: NaOAc, EtOH, RT, 90% [134]; (**iii**) dry toluene, reflux, 1–24 h, 44%–75%. Reproduced in part from [130,131,132,133] with permission of the Royal Society of Chemistry and the American Chemical Society.

Vallance *et al.* [130] identified pentafluorophenyl (PFP) sulfonate esters **61** as nonspecific *Pa*DDAH and bacterial arginine deaminase (ADI) inhibitors, after highlighting their structural similarity with the cysteine protease family of enzymes. This class of inhibitor can be obtained in moderate to good yields (44%–75%) via the 1,3-dipolar cycloaddition between nitrone **60** and the electron deficient vinyl-appended PFP sulfonate **57** (Scheme 13) [131]. The shelf-stable PFP vinylsulfonate **57** is synthesized in moderate yield (59%) from pentafluorophenol (**55**) and 2-chloroethane-1-sulfonyl chloride (**56**) [132], while various nitrones **60** can be generated from their corresponding aldehydes **58** by reaction with *N*-methyl hydroxylamine (**59**) under mild conditions and in good yield (for examples, please see [133,134]).

The electron deficiency of the vinyl PFP sulfonate dipolarophile **57** affords the reversed regioselective 4C-substituted adduct of the isoxazolidine in compound **61**, contrary to the 5C-substituted adduct typically observed with olefins. Although this was previously observed for electron deficient dipolarophiles [135,136,137,138], Caddick *et al.* [131] reported the 4C-substituted isoxazolidine **61** is increasingly favoured at higher temperatures. Each of the nitrones **60** reported by Caddick *et al.* [131] participates in cycloaddition with the vinyl PFP sulfonate **57**, including *C*-aryl- and *C*-alkyl-*N*-methyl species with both electron-donating and electron-withdrawing substituents in addition to poly- and hetero-aromatic substituents. This robust synthetic methodology provides the ability to generate a structurally diverse library of PFP sulfonate esters **61** with moderate ease and high yields [130]. Several PFP sulfonate esters **61** have been screened for their activity as *Pa*DDAH and ADI inhibitors at two concentrations (500 μM and 50 μM), with five compounds emerging as relatively potent *Pa*DDAH inhibitors (IC_50_ = 16–58 μM, see [130] for structures). Vallance *et al.* revealed the mode of inhibition was competitive utilising time-dependence and reversibility experiments [130]. Interestingly, the PFP sulfonate esters **61** have been proposed as potential antibacterial agents, however no data has been reported identifying if *Pa*DDAH inhibition modulates bacterial growth or viability. Likewise, no studies utilizing human DDAH have been reported identifying whether the PFP sulfonate esters exhibit specificity for bacterial *Pa*DDAH. Non-specific PFP sulfonate ester binding to both human DDAH and *Pa*DDAH would be counterproductive for their proposed application as antibiotics.

#### 4.2.2. Indolylthiobarbituric Acid Derivatives

The indolylthiobarbituric acid derivatives **68** [139] were identified as *Pa*DDAH inhibitors by a combination of virtual screening using a compound library comprising 308,000 structures and subsequent biological screening. Among the 109 highest scoring compounds, 90 were commercially available and used in colorimetric enzyme activity assays. Three compounds consisting of two chemical scaffolds were identified as potential *Pa*DDAH inhibitors. One of these scaffolds, indolylthiobarbituric acid (**68a**), together with additional compounds **68b**–**c** identified from a similarity search, emerged as promising inhibitors of *Pa*DDAH (IC_50_ = 2–16.9 μM). The most potent inhibitor of this series was SR445 (**68c**, IC_50_ = 2 μM), and no member of this class of compounds showed any human DDAH inhibition [114].

**Scheme 14:**
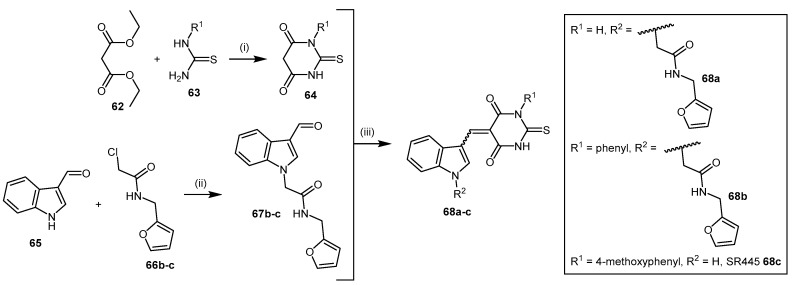
Synthetic scheme for generating indolylthiobarbituric acid derivatives **68**. *Reagents and Conditions*: (**i**) NaOMe, MeOH, 60 °C, 6 h, yields not reported; (**ii**) NaH, DMF, RT, overnight, 83%; (**iii**) 6 M HCl, EtOH, RT, 2–3 h, yields not reported.

This class of inhibitors is comprised of thiobarbituric acid module **64** and indolyl module **67** (Scheme 14). The thiobarbituric acid module **64** is synthesized using a modified version of the method described by Fisher and Mering in 1903, by reaction of *N*-substituted 2-thiourea **63** with diethyl malonate (**62**) [140,141]. The indolyl module **67** is alkylated by deprotonation of indole-3-carbaldehyde (**65**) and reaction with 2-chloro-*N*-furan-2-ylmethyl-acetimide (**66**) using a method previously reported (yield 83%) [142]. The aldehyde substituent of the indolyl derivative **67** is subsequently reacted with the thiobarbituric acid derivative **64** under acidic conditions to afford **68** (yields not reported) [139,143]. This produced a mixture of (*E*)- and (*Z*)-isomers about the linking double bond.

#### 4.2.3. Ebselen

Ebselen (2-phenyl-1,2-benzisoselenazol-3(2*H*)-one, **73**), a selenazole heterocyclic compound with well-known antioxidant properties [144], was recently identified as a potent DDAH-1 inhibitor. This was achieved by utilizing a high throughput screening (HTS) assay of two commercial libraries comprising 2446 compounds [145]. The HTS identified each compound’s ability to inhibit the conversion of the synthetic substrate, *S*-methyl-l-thiocitrulline (SMTC) to citrulline and methanethiol by *Pa*DDAH. Compounds exhibiting *Pa*DDAH inhibitory potentials were validated for their ability to inhibit human DDAH-1 using ADMA as the substrate. The quantification of inhibition was measured using derivatized citrulline formation and colorimetric detection. Characterisation of *Pa*DDAH and human DDAH-1 inhibition by ebselen was subsequently determined by dose-response, reversibility, and ESI-MS experiments.

**Scheme 15:**
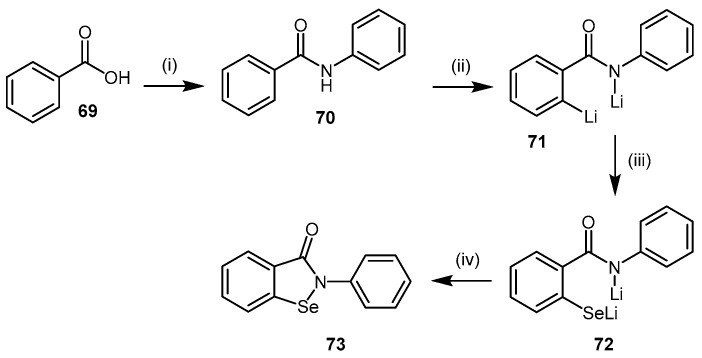
Synthetic scheme for generating ebselen (**73**). *Reagents and Conditions*: (**i**) (1) DCM, pyridine, SOCl_2_, reflux, 2 h; (2) toluene, aniline, reflux, 3 h, 80%; (**ii**) dry THF, *n*-BuLi, nitrogen atmosphere, 0 °C, 30 min, 76% (determined via methylation of **72**); (**iii**) same solution as step (**ii**), Se, 0 °C, 30 min; (**iv**) same solution as step (**iii**), CuBr_2_, 30 min at −78 °C, then 2 h at RT, 63%. Adapted with permission from [147], copyright 1989 American Chemical Society, and [148], copyright 2003 The Pharmaceutical Society of Japan.

While ebselen (**73**) is commercially available, it can be prepared via two main methods [146,147,148]. The method described in Scheme 15 [147,148] is preferred due to its shorter reaction times and elementary design. This method involves the treatment of benzoic acid (**69**) with thionyl chloride and aniline to afford benzanilide (**70**) followed by *ortho*-lithiation to dianion **71** using *n*-butyl lithium. Selenium is then inserted into the carbon-lithium bond of **71**, forming dianion intermediate **72**, whose cyclization to ebselen (**73**) is promoted by copper(II) bromide oxidant.

Ebselen is reported to irreversibly inhibit both *Pa*DDAH (IC_50_ = 96 ± 2 nM) and human DDAH-1 (IC_50_ = 330 ± 30 nM), forming a covalent selenosulfide bond with each enzyme’s respective catalytic cysteine. *In silico* docking experiments suggest inhibitor binding is supported by π-stacking interactions with Phe76 in addition to other polar interactions involving His162. *In vitro* experiments demonstrated ebselen exhibits no effect on human DDAH-1 expression [145].

Ebselen is the most potent DDAH inhibitor with ‘non-substrate-like’ structure reported in the literature to date and demonstrates an anti-inflammatory response [149]. However, its application as a DDAH inhibitor is limited due to its poor DDAH selectivity. A wide range of molecular targets are modulated by ebselen including: NADPH oxidase 2 [150,151], H^+^/K^+^ ATPase transporter [152], Ca^2+^- and phospholipid-dependent protein kinase C [151], lipoxygenase [153], thioredoxin reductase [154], and metallothionein [155]). Moreover, cellular toxicity in leukocytes and hepatocytes has been reported, further reducing the likelihood of ebselen’s use in clinical practice [156,157].

#### 4.2.4. 4-Hydroxy-2-Nonenal

**Scheme 16:**
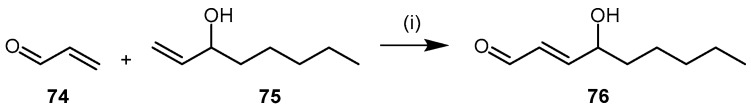
Synthetic scheme for generating 4-hydroxy-2-nonenal (4-HNE, **76**). *Reagents and Conditions*: (**i**) dry oxygen-free DCM, RT, 25 h, 75%, Hoveyda-Grubbs 2nd generation (0.025 eq.).

4-Hydroxy-2-nonenal (4-HNE, **76**) is a cytotoxic aldehyde generated from lipid peroxidation during inflammation [158,159] and is abundant in the body. 4-HNE is highly reactive toward proteinaceous nucleophilic groups, such as thiol and imidazole moieties. Nucleophilic attack affords Michael adducts, whilst Schiff bases are formed with primary amines (e.g., lysine residues) [160,161,162,163]. Different methods have been reported for the synthesis of 4-HNE (**76**) and its derivatives [164,165,166]. The method reported by Soulér *et al.* [166] is described here due to its simplicity, speed, and versatility (Scheme 16). The synthesis is completed in a single cross-metathesis reaction between octen-3-ol (**75**) and 2-propenal (**74**) in the presence of Hoveyda-Grubbs 2nd generation ruthenium catalyst under a nitrogen atmosphere and in dry oxygen-free solvent (Scheme 16). The reaction proceeds at room temperature for 25 h affording the (*E*)-isomer of 4-HNE (**76**) in 75% yield.

4-HNE is reported to react with cysteine and histidine residues and it was this reactivity that led Forbes *et al.* to investigate its use as a human DDAH-1 inhibitor [167]. In Forbes’ experiments, DDAH activity was measured via both colorimetric and radioisotope activity assays, with 4-HNE showing dose-dependent inhibition (IC_50_ = 50 μM) and near complete inhibition at 500 μM. Mass spectrometry experiments elucidated the mechanism of inhibition, whereby the formation of a Michael adduct between 4-HNE and His173 of the DDAH-1 active-site occurs.

#### 4.2.5. PD 404182

6*H*-6-Imino-(2,3,4,5-tetrahydropyrimido)[1,2-*c*]-[1,3]benzothiazine (PD 404182, **79**), is a commercially available heterocyclic pyrimidobenzothiazine and a suitable lead compound for SAR studies due to its simple synthetic methodology [168]. PD 404182 (**79**) is a compound listed in the Library of Pharmacologically Active Compounds (LOPAC) and is reported to have antibacterial [168,169] and antiviral [170,171] activity. Ghebremariam *et al.* [61] utilized a colorimetric assay to screen a library of over 1200 compounds for their ability to inhibit the conversion of ADMA to l-citrulline and dimethylamine by recombinant human DDAH-1. Compounds with reasonable inhibitory potentials were subjected to kinetic characterisation via fluorimetric assay using the synthetic substrate, *S*-methylthiocitrulline. Reversibility and ESI-MS studies were additionally undertaken. PD 404182 was subsequently evaluated for its ability to reduce angiogenesis and to protect against lipopolysaccharide-induced NO production. PD 404182 (**79**) was found to be a potent irreversible competitive human DDAH-1 inhibitor (IC_50_ = 9 μM) and showed promise as an anti-inflammatory and antiangiogenic agent.

**Scheme 17:**
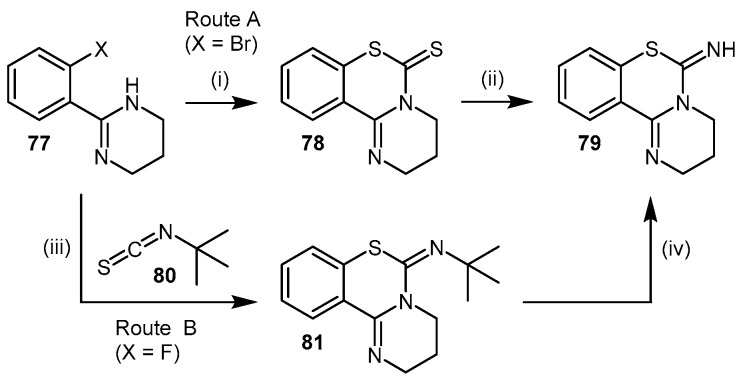
Synthetic scheme for generating PD 404182 (**79**). *Reagents and Conditions*: Route A: (**i**) DMF, NaH, CS_2_, argon atmosphere, 80 °C, 12 h, 88%; (**ii**) (1) 0.1 M NaOH, MeOH/H_2_O (9:1), reflux, 12 h; (2) anhydrous EtOH, BrCN, argon atmosphere, reflux, 2 h, 61% (two steps). Route B: (**iii**) DMF, NaH, argon atmosphere, 80 °C, 2 h, 62%; (**iv**) TFA, CHCl_3_, molecular sieves 4 Å, reflux, 1 h, 85%. Adapted with permission from [172,173]. Copyright 1975, 2010 American Chemical Society.

The synthesis of PD404182 can be achieved via two routes [172,173]. Both approaches generate reasonable to high yields (61%–88%) over several synthetic steps. The first approach involves the reaction of 2-(2′-haloaryl)-tetrahydropyrimidine **77** with carbon disulfide in the presence of sodium hydride, followed by hydrolysis of the carbamodithioate derivative **78** and subsequent treatment with cyanogen bromide to afford **79** (Scheme 17; Route A). Alternatively, the 2-(2′-haloaryl)-tetrahydropyrimidine **77** can be reacted with *tert*-butylisothiocyanate (**80**) in the presence of sodium hydride to afford intermediate **81**, followed by treatment with trifluoroacetic acid to afford **79** (Scheme 17; Route B).

#### 4.2.6. Proton Pump Inhibitors

**Figure 3:**
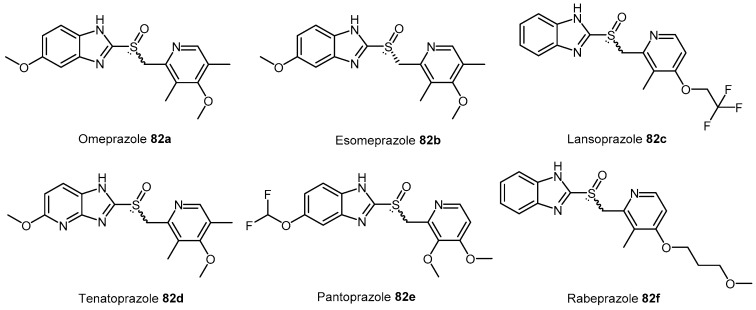
Chemical structures of commonly prescribed proton pump inhibitors (PPIs) **82a**–**f**.

Proton H^+^/K^+^ ATPase pump inhibitors (PPIs) are widely prescribed to inhibit gastric acid secretion and are characterized by a 2-pyridylmethylsulfinylbenzimidazole scaffold. The chemical structures of the commonly prescribed PPIs **82a**–**f** are shown in Figure 3.

HTS experiments utilising a library of 130,000 compounds and recombinant enzyme identified PPIs as human DDAH-1 inhibitors [174,175]. Initial screening was undertaken using a colorimetric assay with ADMA as the substrate. Further characterization of compounds of interest was achieved via a fluorimetric assay using *S*-methylthiocitrulline [174,175]. Surface plasmon resonance (SPR) was additionally utilized to detect the binding of PPIs to amine-coupled DDAH-1 (CM5 sensor chip) [175]. All commercially available PPIs were found to reversibly inhibit DDAH-1 (IC_50_ = 51–63 μM). Interestingly, *in vitro* experiments using human endothelial cells and *in vivo* experiments in mice demonstrated that PPIs increase ADMA concentrations [175] further supporting the evidence that prolonged use of PPIs is associated with an increased risk of cardiovascular disease [176,177].

**Scheme 18:**
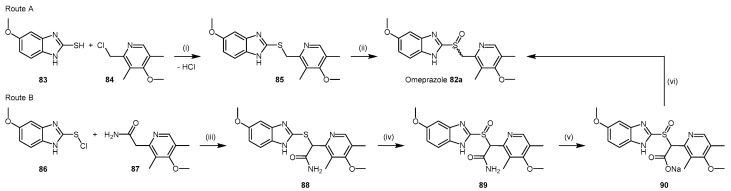
Synthetic scheme for generating omeprazole (**82a**). *Reagents and Conditions*: Route A: (**i**) 1 M NaOH (2 eq.), EtOH, RT, 8 h; (**ii**) catalyst: V_2_O_5_, NaVO_3_, NH_4_VO_3_, [(CH_3_COCH_2_COCH_3_)_2_VO] (0.0001–0.1 eq.), H_2_O_2_: 10%–70% aqueous or organic solution (1–3 eq.), solvent: halogenated hydrocarbons, ethers, alcohols, ketones, nitriles, or H_2_O, temperature: 0 °C to boiling point of the solvent, 0.5–24 h, 89%–93%. Route B: (**iii**) condensation, conditions not specified in patent; (**iv**) [(CH_3_COCH_2_COCH_3_)_2_VO] (0.006 eq.), 30% H_2_O_2_, acetone, 0–5 °C for 1 h then 20–22 °C for 1 h, 90%; (**v**) 10% NaOH, nitrogen atmosphere, 50 °C, 3 h; (**vi**) CO_2_, extraction into 1:1 *v*/*v* isopropanol/toluene, reflux, 20–30 min, 42% (across steps (**v**) and (**vi**)).

PPIs are comprised of pyridine and benzimidazole moieties with different substituents. Their syntheses are protected by current patents (with the exception of omeprazole (**82a**)). The original patent for omeprazole (**82a**) [178] details preparation of the 2-(chloromethyl)pyridine derivative **84**. Condensation [179] of compound **84** with 2-mercaptobenzimidazole derivative **83** affords the thioether precursor **85**, which is subsequently oxidized to the sulfoxide in omeprazole (**82a**) using conditions such as hydrogen peroxide and a vanadium catalyst [180] (Scheme 18; Route A).

Further development of the synthetic method to yield omeprazole (**82a**) [180] identified that the coupling between the pyridine and benzimidazole moieties can be modified to simplify purification. Principally, this method avoids the formation of the thioether intermediate **85**. Furthermore, the crude product containing omeprazole (**82a**) produced from the original synthesis is prone to discolouration during the oxidation step [180]. The revised synthesis (Scheme 18; Route B) proceeds via condensation of 2-chloromercaptobenzimidazole derivative **86** with a primary amide pyridine derivative **87** to afford the acetamide thioether intermediate **88**, which is oxidized to the acetamide sulfoxide **89**. Compound **89** is subsequently hydrolysed to the carboxylic acid salt **90** under alkaline conditions, with decarboxylation yielding omeprazole (**82a**) or lansoprazole (**82c**), depending on the benzimidazole **86** and the pyridine **87** derivatives used as the starting materials.

#### 4.2.7. 1,2,3-Triazoles

**Scheme 19:**
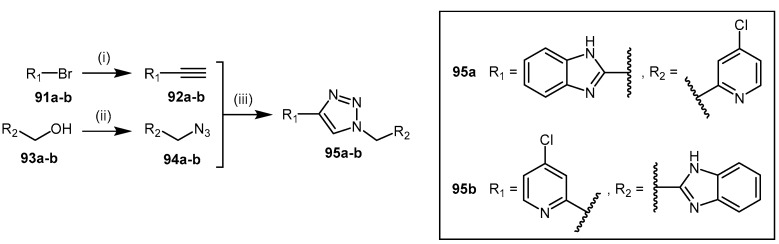
Synthetic scheme for generating 1,2,3-triazoles **95**. *Reagents and Conditions*: (**i**) **92a** (Ph_3_P)_2_PdCl_2_, CuI, TEA, TIPS-acetylene, DMF, 80 °C, 72 h, 30%, **92b** (Ph_3_P)_2_PdCl_2_, CuI, TEA, ethynyl-TMS, 80 °C, 20 h, 48%; (**ii**) DPPA, DBU, toluene, RT, 16 h, **94a** 55%, **94b** 83%; (**iii**) CuI, sodium ascorbate, TEA, DCM, RT, 24 h, **95a** 65%, **95b** 30%. Reproduced in part from [117] with permission of the Royal Society of Chemistry.

The synthesis of 1,2,3-triazoles **95** (Scheme 19) comprised of 4-halopyridine and benzimidazole units have been explored as ‘non-substrate-like’ DDAH-1 inhibitors [117]. The synthesis of these derivatives was undertaken based on reports that identified binding of both the 4-halopyridine and benzimidazole fragments to human DDAH-1 [181]. A triazole linker was selected to connect these fragments via copper(I)-catalysed ‘click’ cycloaddition between the terminal alkyne **92** and azide **94** (Scheme 19). The alkyne **92** is synthesized by Sonogashira coupling of the alkyne derivative with the required halogenated species **91** [182,183]. The azide **94** is synthesized by reaction of diphenylphosphoryl azide with the required primary alcohol **93** [184].

Human DDAH-1 inhibition, albeit low (IC_50_ = 422 μM, 57%, inhibition at 1 mM), was reported with triazole **95a**. In general, **95a**–**b** failed to show significant inhibitory potential and may be due to the triazole linker not facilitating a productive inhibitory binding conformation of the fragments in the enzyme active-site.

## 5. Conclusions

Current evidence suggest that inhibiting the DDAH family of enzymes may be used as a therapeutic tool to indirectly inhibit the NOS enzymes via increasing concentrations of endogenous NOS inhibitors, such as ADMA and l-NMMA. Therefore the development and synthesis of DDAH inhibitors is emerging as a promising field of clinical research. Furthermore, engineering compounds that target bacterial DDAH may represent a suitable strategy for the development of new antimicrobial agents; however further investigations are required. This has led to different classes of DDAH inhibitors being reported within the last decade characterized by alternative features in terms of chemical structure, potency, enzyme selectivity, and mechanism of action. The importance placed on each of these features depends on the end application of the inhibitor. Table 3 summarizes the most potent DDAH inhibitors documented in the literature to date. Some compounds, namely PFP sulfonate esters **61** or indolylthiobarbituric acid derivatives **68**, exhibit significant *Pa*DDAH inhibition. In particular, selectivity experiments using indolylthiobarbiturates failed to exhibit inhibitory effects on the human isoenzyme, therefore derivatives of this structural class are well positioned for further investigation in antibiotic applications. Among classes of molecules which are active on the human isoenzyme (amino acid derivatives **27**, **29**, **39**, **40**, **44**, **49**, **54**, ebselen (**73**), 4-HNE (**76**), PD 404182 (**79**), PPIs **82**, 1,2,3-triazoles **95**), L-257 (**39**) and L-291 (**40**) are highly selective for DDAH-1, while ornithine derivatives such as L-VNIO (**54a**) or L-IPO (**54b**) are known to inhibit not only DDAH, but also arginase and the NOS family of enzymes. Furthermore, compounds such as ebselen (**73**) and 4-HNE (**76**), although very potent DDAH inhibitors, are well known to exhibit a range of off-target effects, thus are more suitable for experiments *in vitro* rather than *in vivo*.

In terms of the varied approaches to chemical synthesis of the DDAH inhibitors discussed here, many can be synthesized in moderate to excellent overall yields and use reaction sequences that can be easily modified to enable the design of a large number of analogues. The commercial availability of several of the compounds determined to be DDAH inhibitors (2-chloroacetamidine (**11**), ebselen (**73**) 4-HNE (**76**) and the PPIs **82**) demonstrates the versatility of some of the synthetic routes for large scale production.

Based on the *in vitro* pharmacokinetic and/or enzyme selectivity data compiled in this review, there appear to be three promising human DDAH-1 inhibitors that have the potential to progress to clinical trial: ZST316 (*K*_i_ = 1 μM), Cl-NIO (*K*_i_ = 1.3 μM), and L-257 (*K*_i_ = 13 μM) (Table 3). Despite exhibiting the most potent inhibitory response *in vitro*, a paucity of data exists with respect to the *in vivo* pharmacokinetic and pharmacodynamic profiling of ZST316. Literature reports involving the enzyme selectivity of Cl-NIO shows considerable promise, with no measurable cross-reactivity in experiments specifically involving eNOS [129]. However, data are not available regarding this inhibitor‘s interactions with iNOS, nNOS, or the arginase family of enzymes. Contrary to this, data reported for L-257 by Kotthaus *et al.* [115] demonstrates that L-257 is highly selective for DDAH-1. The administration of L-257 in a rodent model of septic shock not only improved survival, but additionally improved haemodynamic and organ function [67], indicating a potential clinical role for L-257.

Interestingly, little is understood about the regulation and pharmacological modulation of human DDAH-2. As such, the development of improved metabolomic approaches for the sensitive measurement of DDAH-2 activity *in vitro* is an important step forward in profiling the endogenous substrates and biochemical role(s) of this important enzyme in humans.

The continued exploration of DDAH inhibition and the synthesis of new chemical entities with improved pharmacokinetic parameters are therefore at an exciting junction in the development of novel medicines.

## Figures and Tables

**Table 1:** Reported inhibitory constants for 2-chloroacetamidine (**11**) [106].

Enzyme	*K*_i_ (mM)	*K*_inact_ (min^−1^)
*Pa*DDAH	3.1 ± 0.8	1.2 ± 0.1
PAD4	20 ± 5	0.7 ± 0.1

**Table 2:** Inhibitory parameters for the *N*-monosubstituted l-arginine analogues with varied *N*-substituents (R groups) [114,117]. 
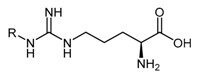

Entry	R–	Inhibition at 1 mM (%)	IC_50_ (μM)	*K*_i_ (μM)
1 ^1^	* CH_3_OCH_2_CH_2_–	83	31	13
2 ^1^	CH_3_NHCH_2_CH_2_–	29	-	-
3 ^1^	(CH_3_)_2_NHCH_2_CH_2_–	14	-	-
4 ^1^	CH_3_SHCH_2_CH_2_–	80	408	-
5 ^1^	FCH_2_CH_2_–	67	379	-
6 ^1^	BnCH_2_–	55	866	-
7 ^2^	CH_3_CH_2_CH_2_–	60	283	90
8 ^2^	CH_2_CHCH_2_–	70	207	58
9 ^2^	CH_2_CHCH_2_CH_2_–	71	189	57
10 ^2^	CHCCH_2_–	83	55	17
11 ^2^	F_3_CCH_2_–	41	-	-
12 ^2^	O_2_N–	6	-	-
13 ^2^	NH_2_COCH_2_CH_2_–	43	-	-

* Denotes L-257 (**39**) (Scheme 9); ^1^ Measured by Ultra Performance Liquid Chromatography-Mass Spectrometry; ^2^ Measured by colorimetric assay.

**Table 3:** Summary of the main DDAH inhibitors and their kinetic parameters.

Chemical Number	Chemical Name	Chemical Structure	Enzyme	IC_50_ (µM)	*K*_i_ (µM)	*K_inact_* (min^−1^)	Reference
(**9**)	*S*-Nitroso-l-homocysteine (HcyNO)	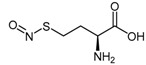	Bovine DDAH-1	75	690	0.38	[105]
(**11**)	2-Chloroacetamidine	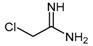	*Pa*DDAH ^1^	-	3100	1.2	[106]
(**18**)	*N*-But-3-ynyl-2-chloro-acetamidine	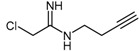	Human DDAH-1	350	-	-	[109]
(**21**)	(*S*)-2-Amino-4-(3-methylguanidino)butanoic acid (4124W)	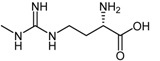	Rat DDAH-1	416	-	-	[110]
(**39**)	*N^G^*-(2-Methoxyethyl)-l-arginine (L-257)	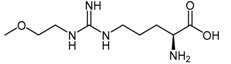	Human DDAH-1	31 ^2^	13 ^2^	-	[112]
(**40**)	*N^G^*-(2-Methoxyethyl)-l-arginine methyl ester (L-291)	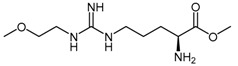	Rat DDAH-1	20	-	-	[112]
(**44a**)	2-Amino-5-(3-(2-methoxyethyl)guanidino)-*N*-(methylsulfonyl)-pentanamide (ZST316)	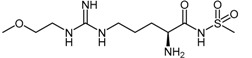	Human DDAH-1	3	1	-	[117]
(**54a**)	*N*^5^-(1-Imino-3-butenyl)-l-ornithine (L-VNIO)	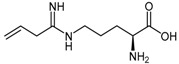	Human DDAH-1	13	2	-	[114]
(**54b**)	*N*^5^-(1-Iminopropyl)-l-ornithine (L-IPO)	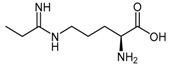	Human DDAH-1	-	52	-	[128]
(**54c**)	*N^5^*-(1-Imino-2-chloroethyl)-l-ornithine (Cl-NIO)	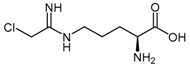	Human DDAH-1	-	1.3	0.34	[129]
**61**	Pentafluorophenyl (PFP) sulfonate esters	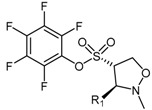	*Pa*DDAH ^1^	16–58	-	-	[130]
**68a–c**	Indolylthiobarbituric acid derivatives	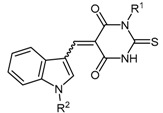	*Pa*DDAH ^1^	2–17	-	-	[139]
(**73**)	Ebselen	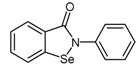	Human DDAH-1	0.33	-	-	[145]
(**76**)	4-Hydroxy-2-nonenal (4-HNE)	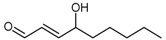	Human DDAH-1	50	-	-	[167]
(**79**)	6*H*-6-Imino-(2,3,4,5-tetrahydropyrimido)[1,2-*c*]-[1,3]benzothiazine (PD 404182)	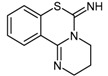	Human DDAH-1	9	-	-	[61]
**82a–f**	Proton H^+^/K^+^ ATPase pump inhibitors (PPIs)	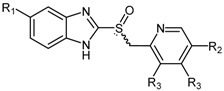	Human DDAH-1	51–63	-	-	[175]

^1^ *Pa*: *Pseudomonas aeruginosa*; ^2^ as determined by Tommasi *et al*. [117].

## Abbreviations

The following abbreviations are used in this manuscript:

DDAH	Dimethylarginine dimethylamino hydrolase
ADMA	Asymmetric dimethylarginine
L-NMMA	Monomethyl arginine
NOS	Nitric oxide synthase
NO	Nitric oxide
SDMA	Symmetric dimethylarginine
PRMTs	Protein arginine methyl transferases
CAT	Cationic amino acid transporter
AGXT2	Alanine-glyoxylate amino transferase 2
PKA	Protein kinase A
Akt	Protein kinase B
VEGF	Vascular endothelial growth factor
iNOS	Inducible nitric oxide synthase
TNF-α	Tumour necrosis factor α
LDL	Low density lipoprotein
IL-1β	Interleukin-1β
AT_1_-R	Type 1 angiotensin receptor
DNA	Deoxyribonucleic acid
FXR	Farnesoid X receptor
HcyNO	*S*-Nitroso-l-homocysteine
NaNO_2_	Sodium nitrite
NaOH	Sodium hydroxide
HCl	Hydrochloric acid
Min	Minutes
*K*i	Inhibition constant
µM	Micromolar
H_2_O	Water
Cys	Cysteine
His	Histidine
*Pa*DDAH	*Pseudomonas aeruginosa* dimethylarginine dimethylamino hydrolase
PAD	Peptydilarginine deaminase
NaOMe	Sodium methoxide
MeOH	Methanol
AcOH	Acetic acid
NH_4_Cl	Ammonium chloride
PEO	Poly(ethylene oxide)
DCM	Dichloromethane
TEA	Triethylamine
DMAP	4-Dimethylaminopyridine
MsCl	Methanesulfonyl chloride
Et_2_O	Diethyl ether
RT	Room temperature
NaN_3_	Sodium azide
DMF	*N*,*N*-Dimethylformamide
Ph_3_P	Triphenylphosphine
4124W	(*S*)-2-Amino-4-(3-methylguanidino)butanoic acid
Boc	*tert*-Butyloxycarbonyl
M	Molar
DEAD	Diethyl azodicarboxylate
THF	Tetrahydrofuran
DIPEA	*N*,*N*-Diisopropylethylamine
CH_3_CN	Acetonitrile
SOCl_2_	Thionyl chloride
SAR	Structure activity relationship
IC_50_	Inhibition constant
L-257	*N*^G^-(2-Methoxyethyl)-l-arginine
L-291	*N*^G^-(2-Methoxyethyl)-l-arginine methyl ester
Orn	Ornithine
Bu	Butyl
Z	Carboxybenzyl
TFMSA	Trifluoromethanesulfonic acid
TFA	Trifluoroacetic acid
CDI	1,1′-Carbonyldiimidazole
DBU	1,8-Diazabicyclo[5.4.0]undec-7-ene
h	Hours
HATU	1-[Bis(dimethylamino)methylene]-1*H*-1,2,3-triazolo[4,5-*b*]pyridinium 3-oxide hexafluorophosphate
mM	Millimolar
DMSO	Dimethyl sulfoxide
*i*-BuCOCl	Isobutyl chloroformate
NH_4_OH	Ammonium hydroxide
TFAA	Trifluoroacetic anhydride
ZnBr_2_	Zinc bromide
NH_2_OH.HCl	Hydroxylamine hydrochloride
NaHCO_3_	Sodium bicarbonate
L-VNIO	*N*^5^-(1-Imino-3-butenyl)-l-ornithine
EtOH	Ethanol
EtOAc	Ethyl acetate
L-IPO	*N*^5^-(1-Iminopropyl)-l-ornithine
Cl-NIO	*N*^5^-(1-Imino-2-chloroethyl)-l-ornithine
PFP	Pentafluorophenyl
NaOAc	Sodium acetate
ADI	Arginine deaminase
HTS	High throughput screening
SMTC	*S*-methyl-l-thiocitrulline
ESI-MS	Electrospray ionization mass spectrometry
BuLi	Butyl lithium
Se	Selenium
CuBr_2_	Copper(II) bromide
NADPH	Nicotinamide adenine dinucleotide phosphate
H+/K+ ATPase	Proton/potassium ATPase
Ca^2+^	Calcium 2+ cation
4-HNE	4-Hydroxy-2-nonenal
LOPAC	Library of Pharmacologically Active Compounds
NaH	Sodium hydride
CS_2_	Carbon disulfide
BrCN	Cyanogen bromide
Å	Angstrom
PPIs	Proton pump inhibitors
SPR	Surface plasmon resonance
CM	Carboxymethylated
(Ph_3_P)_2_PdCl_2_	Bis(triphenylphosphine)palladium chloride
CuI	Copper(I) iodide
TIPS	Triisopropylsilyl
TMS	Trimethylsilane
DPPA	Diphenylphosphoryl azide
